# *Mycobacterium tuberculosis* CysA2 is a dual sulfurtransferase with activity against thiosulfate and 3-mercaptopyruvate and interacts with mammalian cells

**DOI:** 10.1038/s41598-019-53069-6

**Published:** 2019-11-14

**Authors:** A. N. Meza, C. C. N. Cambui, A. C. R. Moreno, M. R. Fessel, A. Balan

**Affiliations:** 1 Department of Microbiology, Institute of Biomedical Sciences, Applied Structural Biology Laboratory, LBEA, University of São Paulo, São Paulo, SP Brazil; 2 Institute of Biology, Post-graduate Program in Genetics and Molecular Biology, University of Campinas, UNICAMP, Campinas, SP Brazil; 3 Department of Microbiology, Vaccine Development Laboratory, Biomedical Sciences Institute, University of São Paulo, São Paulo, SP Brazil

**Keywords:** Enzyme mechanisms, Cellular microbiology, X-ray crystallography

## Abstract

Cyanide is a toxic compound that is converted to the non-toxic thiocyanate by a rhodanese enzyme. Rhodaneses belong to the family of transferases (sulfurtransferases), which are largely studied. The sulfur donor defines the subfamily of these enzymes as thiosulfate:cyanide sulfurtransferases or rhodaneses (TSTs) or 3-mercaptopyruvate sulfurtransfeases (MSTs). In *Mycobacterium tuberculosis*, the causative agent of tuberculosis, the gene Rv0815c encodes the protein CysA2, a putative uncharacterized thiosulfate:cyanide sulfurtransferase that belongs to the essential sulfur assimilation pathway in the bacillus and is secreted during infection. In this work, we characterized the functional and structural properties of CysA2 and its kinetic parameters. The recombinant CysA2 is a α/β protein with two rhodanese-like domains that maintains the functional motifs and a catalytic cysteine. Sulfurtransferase activity was determined using thiosulfate and 3-mercaptopyruvate as sulfur donors. The assays showed K_m_ values of 2.89 mM and 7.02 mM for thiosulfate and 3-mercaptopyruvate, respectively, indicating the protein has dual activity as TST and MST. Immunological assays revealed that CysA2 interacted with pulmonary cells, and it was capable to activate macrophages and dendritic cells, indicating the stimulation of the immune response, which is important for its use as an antigen for vaccine development and immunodiagnostic.

## Introduction

*Mycobacterium tuberculosis* is the causative agent of the tuberculosis, one of the most common life-threatening infectious diseases worldwide, which effective treatment is still under concerning^1,2^. Cysteine biosynthesis is a crucial metabolic pathway for the pathogen, not only by generating amino acid for the *de novo* synthesis of proteins, but by favoring reduced thiol production as an oxidative defense component, which is particularly vital in the dormant stage of *M. tuberculosis*^3^. The sulfur assimilation pathway in *M. tuberculosis* involves the sulfate uptake system SubICysTWA1, an ATP-Binding Cassette (ABC) transporter, and the sequential activity of a set of enzymes (CysCDNHI) that will generate L-cysteine for further synthesis of methionine, mycothiol and cofactors^4^. Since this pathway is only present in bacteria, it becomes an important mechanism to target aiming the inhibition of bacterial oxidative stress, host infection and establishment of long-term infections^5,6^.

The genome analysis of *M. tuberculosis* showed four genes encoding proteins with rhodanese (TST) domains: *cysA2* (Rv0815c), *cysA3* (Rv3117), *sseA* (Rv3283) and *sseB* (Rv2291). SseA and SseB share, respectively, 50% and 26% of amino acid sequence identity compared to CysA2, however they are not directly involved with the same metabolic pathway of CysA2. On the other hand, CysA2 and CysA3 are identical proteins that reveal duplicity in the genome and this redundancy suggests an important role in the bacillus metabolism^7^. Despite the availability of CysA2 three-dimensional structure in the Protein Data Bank (reference code 3HWI) and one study showing its potential as a serodiagnostic biomarker for *M. tuberculosis* infection^8^, no characterization of the enzymatic activity is known. Moreover, CysA2, that is a cytoplasmic protein, was also identified in samples of secreted fractions gradually released during growth of the bacillus *M. tuberculosis*^9–12^.

Sulfurtransferase enzymes, specifically thiosulfate:cyanide sulfurtransferases, also known as rhodaneses (EC 2.8.1.1), catalyze the conversion of cyanide and thiosulfate into sulfite and thiocyanate, while 3-mercaptopyruvate sulfurtransferases (MSTs, EC 2.8.1.2) use 3-mercaptopyruvate as sulfur donor to cyanide to obtain pyruvate and thiocyanate as final products^13^. The analysis of the rhodanese sequences shows two characteristic signatures [F/Y]X3-H-[L/I/V]-PGA-X2-[L/I/V/F] and [A/V]-X2-[F/Y]-[D/E/A/P]-G-[G/S/A]-[W/F]Y/W], respectively, located in the N- and C-termini regions^12^. In addition, sulfurtransferases can be structurally classified into four different groups: (I) single domain proteins, (II) tandem domain proteins, (III) multidomain proteins, and (IV) proteins with the elongated loop in the active site. TSTs and MSTs belong to group II of tandem domain proteins that is formed by two rhodanese domains, one being active and the other inactive^12^.

The best-characterized sulfurtransferase TST is the bovine rhodanese (Rhobov), which represents the reference structure for the rhodanese subfamily^12^. The rhodaneses of bovine (Rhobov) and *Azotobacter vinelandii* (RhdA)^14^ exhibit 22% sequence identity, and their crystal structures display very similar three-dimensional conformations. The *Escherichia coli* GlpE, a sulfurtransferase TST, is composed by a single rhodanese domain and displays catalytic activity, evidencing that the inactive N-domain is not important to perform the catalysis in this microorganism^15^. *Pseudomonas aeruginosa* (RhdA)^16^ and *Azotobacter vinelandii* (RhdA) belong to the same eubacterial family, but only *P. aeruginosa* is capable to produce cyanide. In *Trichoderma spp*. and some strains of *Fusarium spp*., the presence of rhodaneses was described but showing higher affinity of the enzyme (lower Km) towards cyanide than the found in literature for orthologues^17^. Similarly to rhodaneses, MSTs are also considered to perform the cyanide detoxification, but their role and the mechanism of action are poorly characterized^18^. Mercaptopyruvate sulfurtransferases were first described in rat liver^19,20^, and lately studied in *Arabidopsis thaliana*^21^, *E. coli*^22^, *Leishmania spp*.^17^ and *Homo sapiens*^23^.

In this work, we carried out the first biophysical characterization of CysA2 and showed that the enzyme is active as a dimer and it is capable to use both thiosulfate and 3-mercaptopyruvate as sulfur donor substrates. In addition, we explored the characterization of its immunogenic properties showing that CysA2 can bind to lung cells (TC-1) of C57BL/6 mice, and also to activate murine macrophages J774 and spleen dendritic cells.

The data presented in this work show the first structural and functional characterization of a protein from *M. tuberculosis* that has high potential to be target for development of inhibitors for vaccine development and tuberculosis diagnosis. The ability to use two different substrates is unprecedented for this kind of enzyme and arises questions about its role *in vivo*.

## Results

### *M. tuberculosis cysA2* gene encodes one of four putative thiosulfate sulfurtransferases found in the bacillus genome

**Figure 1:**
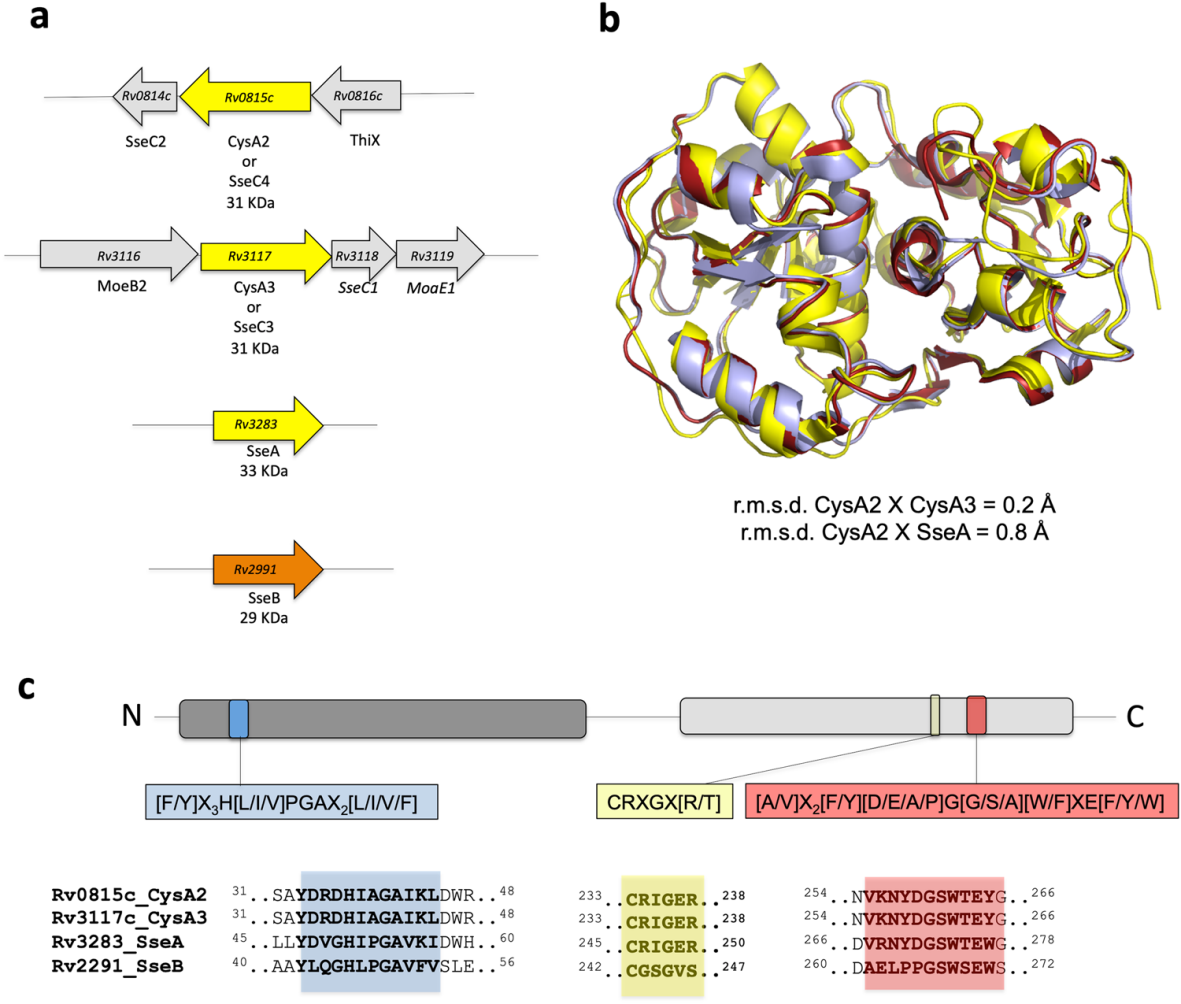
Genome organization of *cysA2* and related genes, structural conservation and sulfurtransferase motifs in the correspondent proteins. (**a**) Genome organization of *cysA2* and the three other putative sulfurtransferases. *cysA2* and *cysA3* genes are identical and organized in two different operons that also have genes involved in the molybdenum metabolism. Gene codes and the name of the proteins are shown in agreement with the Tuberculist site. The molecular weight of the proteins is shown in kDa. (**b**) Structural superimposition of the three-dimensional structures of *M. tuberculosis* CysA2 (PDB code 3HIW, yellow), CysA3 (PDB code 3AAY, red) and SseA (PDB code 3HZU, blue). **(c)** Amino acid sequence alignment of CysA2 and the three related proteins identified in *M. tuberculosis* detaching the motifs that characterize the sulfurtransferases TSTs and MSTs.

The analysis of the genome of *M. tuberculosis* showed four genes encoding for putative thiosulfate sulfurtransferases, *Rv0815* (CysA2), *Rv3117* (CysA3), *Rv3283* (SseA) and *Rv2991* (SseB) (Fig. 1a). *cysA2* and *cysA3* are located in different regions of the genome but encode identical proteins. Genes related to the metabolism of sulfur and molybdenium are close to *cysA2* and *cysA3*^24^. On the other hand, Rv3283 (*sseA*) and Rv2991 (*sseB*) are isolated in the genomes with putative functions as thiosulfate sulfurtransferases that share 50% and 26% of amino acid sequence identity with CysA2/CysA3, respectively. The superimpositions of the available three-dimensional structures of CysA2, CysA3 and SseA (PDB codes 3HWI, 3AAY, 3HZU, respectively) revealed they have identical folding (Fig. 1b). Moreover, the amino acid sequence alignments showed that from the four sulfurtransferases, only SseB has the MSTs motif **CG(S**/T) (Fig. 1c). Proteomics studies and two-dimensional gel electrophoresis followed by mass spectrometry in standing cultures^25,26^ showed that although there were no differences in the expression levels of genes *cysA2*, *cysA3* and *sseB* in ancient and modern Beijing strains, the average rhodanese activity observed in the cell lysate of ancient Beijing strain was 23% higher than the rhodanese activity observed in the modern one^26^. A mutagenesis analysis using Himar1-based transposon showed the genes are non-essential in *M. tuberculosis* CDC1551 strain^27^. On the other hand, the *M. tuberculosis* H37Rv *sseA* mutant showed slow growth rate^28^ and impairment of survival of the bacillus in primary murine macrophages^29^.

Based on the amino acid sequence alignment, the four proteins show the two signatures of rhodanese enzymes^12^. The sequence [F/Y]-X_3_-H-[L/I/V]-PGA-X_2_-[L/I/V/F] in the N-terminal domain and [A/V]-X_2_-[F/Y]-[D/E/A/P]-G-[G/S/A]-[W/F]-X-E-[F/Y/W] in the C-terminal domain. *M. tuberculosis* CysA2, CysA3 and SseA proteins show the characteristic motif CRIGER [CRXGX(R/T)] of thiosulfate surfultransferases and SseB has a similar motif found in 3-mercaptopyruvate sulfurtransferases [CG(S/T)GVT]^30^ (Fig. 1b).

Based on the available three-dimensional structure of CysA2, we performed a comparison with putative orthologues studied in the literature. A search in the Protein Data Bank for the orthologues revealed the TSTs SseA of *M. tuberculosis* (50%), *E. coli* Rhobov*, Azotobacter vinelandii* RhdA (both with 30%) and *E. coli* YnjE (27%). Moreover, two MSTs that shared around 30% of amino acid sequence identity with CysA2 were identified in *Saccharomyces cerevisiae* (TUM1) and *E. coli* (SseA), respectively (Fig. S1a, Supplementary Material). Despite of the low amino acid sequence conservation, all the proteins maintained the structural α/β folding. The structural alignment of CysA2 showed a very low RMSD (0.5 to 0.6 Å) when compared to TST proteins, and higher values when compared to 3-mercaptopyruvate sulfurtransferases (RMSD from 1.4 to 2.6 Å) (Fig. S1b,c, Supplementary Material).

**Figure 2:**
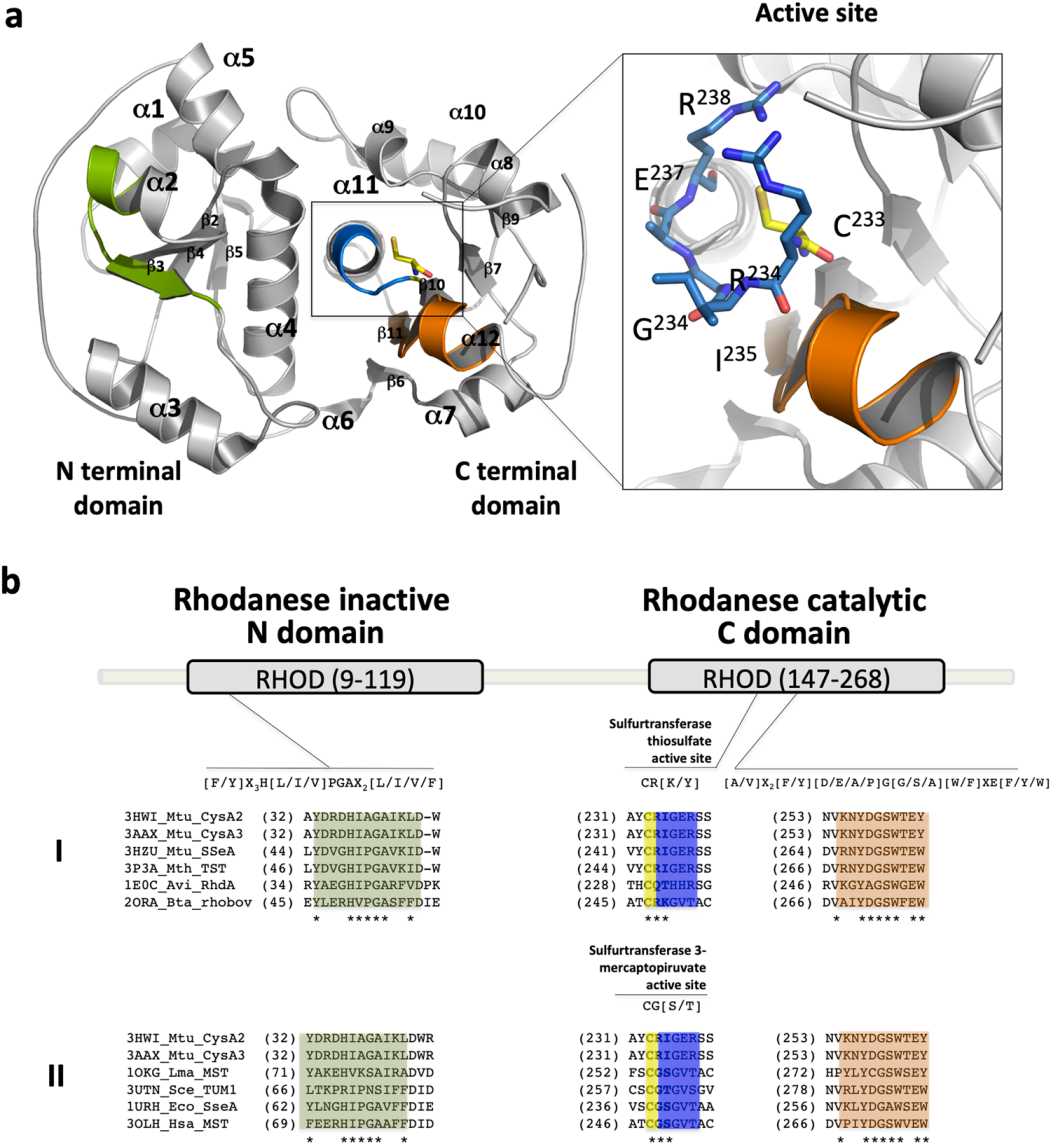
The three-dimensional structure of the *M. tuberculosis* CysA2 (PDB code 3HWI). (**a**) Cartoon representation of CysA2 structure consisting of 12 α-helices and two β-sheets, respectively, with 4 and 5 strands in the N and C terminal domains. The position of the two signatures for rhodaneses, in the N and C domains, are shown in green and orange, respectively. The residues from active site are colored in blue and the catalytic cysteine (C233) in yellow. A detailed view and position of the residues (^233^CRIGER^238^) is also showed in sticks. (**b**) The amino acid sequence of *M. tuberculosis* CysA2 was aligned and compared with TSTs (I) and MSTs (II) described in the literature. The rhodanese signatures present in N (green) and C (orange) domains are conserved in all sequences. The residues of CysA2 active site are closely related to TSTs [CR(K/Y)] and not MSTs CG(S/T). TSTs: 3HWI_Mtu_CysA2: *M. tuberculosis* CysA2, 3AAX_Mtu_CysA3: *M. tuberculosis* CysA3, 3HZU_Mtu_SseA: *M. tuberculosis* SseA, 3P3A_Mth_TST: *Mycobacterium thermoresistibile* TST, 1E0C_Avi_RhdA: *Azotobacter vinelandii* RhdA, 2ORA_Bta: *Bos taurus* Rhobov. MSTs: 1OKG_Lma_MST: *Leishmania major* LmMST, 3UTN_Sce_TUM1: *Saccharomyces cerevisiae* TUM1, 1URH_Eco_SseA: *Escherichia coli* SseA and 3OLH_Hsa_MST: *Homo sapiens* HsMST.

The structure of CysA2 was solved at 2.29 Å and revealed two α/β domains (137 amino acids of the N domain and 130 belonging to the C domain) (Fig. 2a). Based on the TSTs from literature, the N domain is inactive, whereas the C domain is responsible for the catalytic activity. The conserved catalytic cysteine of the active site^31^ (Cys233) is present in *M. tuberculosis* CysA2 structure (Fig. 2a, in yellow sticks) as well as the signature sequences located at the N- and C-terminal domains (Fig. 2b, green and orange boxes, respectively). The residues CRI (amino acids 233–235) form the active site in CysA2, which is more consistent with TST motifs than MSTs (Fig. 2b,I and II).

### CysA2 alternates the dimer/monomer states upon substrates binding

*M. tuberculosis* CysA2 was purified by immobilized nickel affinity chromatography followed by size-exclusion chromatography. According to the analytical gel filtration, the protein eluted as a dimer of 74 kDa (Fig. S2, Supplementary Material). Similarly, when submitted to dynamic light scattering analysis CysA2 showed a hydrodynamic radius (*r*) of 3.8 nm, which was consistent with a dimer. All the experiments were performed just about the purification steps.

**Figure 3:**
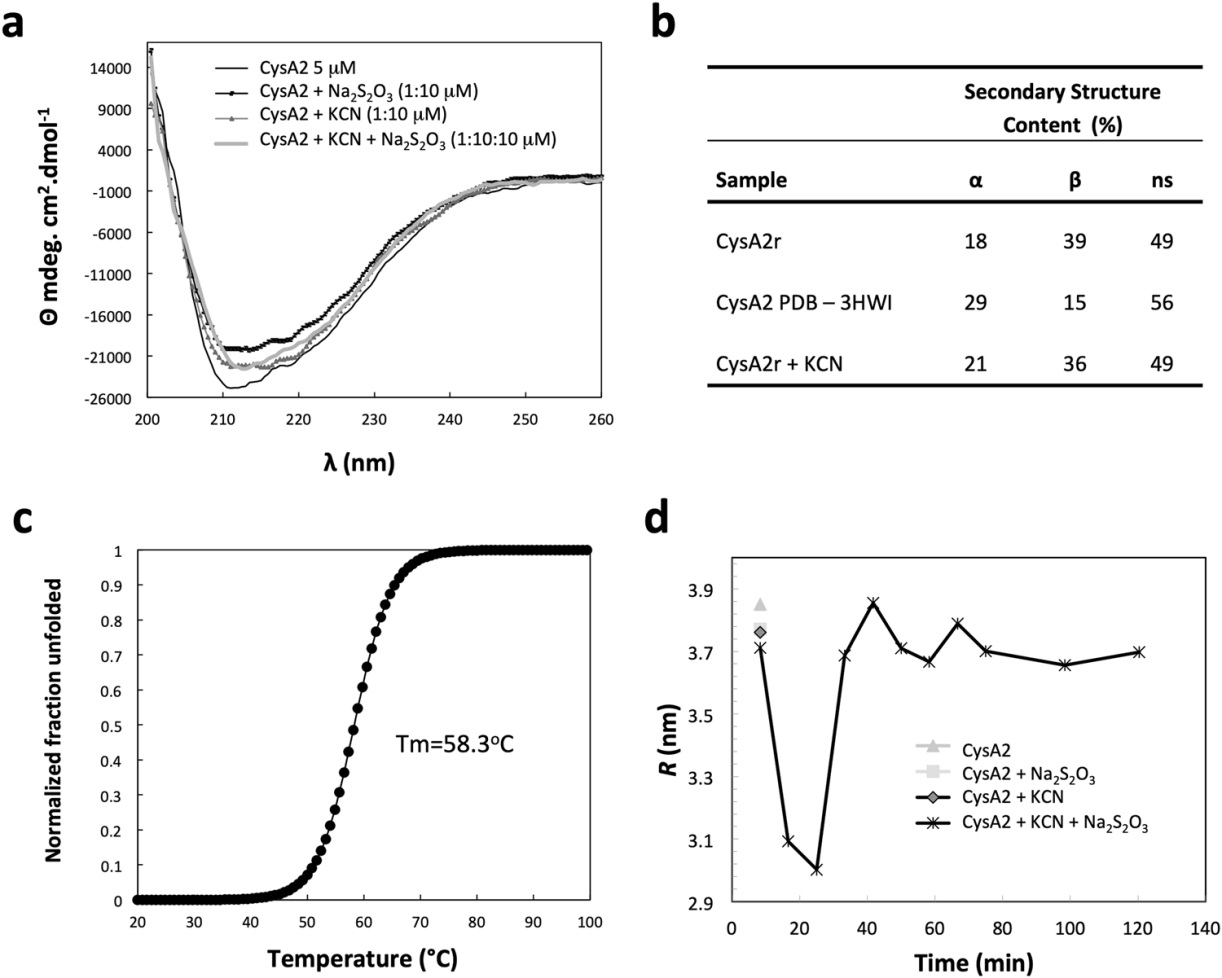
Spectroscopic analysis of *M. tuberculosis* CysA2. (**a**) Circular dichroism spectra of CysA2 in absence and presence of co-substrates cyanide and thiosulfate. (**b**) Comparison of the secondary structure content of the recombinant CysA2 (CysA2r) with the structure deposited in PDB (CysA2 PDB 3HWI) and in presence of KCN (CysA2r + KCN). (**c**) Thermal unfolding of the protein monitored at 222 nm revealing a Tm of 58.3 °C. CysA2 is considered a stable protein. (**d**) Dynamic light scattering analysis of CysA2 in absence and presence of thiosulfate (Na_2_S_2_O_3_), cyanide (KCN) and both. The results show that the substrates induced significant changes in the hydrodynamic radius, which is reduced just after addition of thiosulfate and cyanide (3.8 nm to 3.0), and it is reestablished after 10 minutes.

CysA2 was purified as a structured protein that showed a circular dichroism (CD) spectrum characteristic of α/β folding (Fig. 3a). Deconvolution of the CD data showed a slight difference in the content of α-helices (18% to 21%) and β-sheets (39% to 36%) between the recombinant protein (CysA2r) and the protein in the presence of cyanide (CysA2r + KCN) (Fig. 3b), indicating changes in the structure upon interaction. Compared to the PDB crystal structure, the values of CysA2 samples in solution presented lower α-helices and higher β-sheet contents (29% and 15%, respectively). These differences can be attributed to the significant variation between the protein purification and crystallization conditions. In our experiments, the protein was maintained in absence of salts and 20 mM Tris-HCl pH 8.0 buffer while the crystals were obtained in 25% PEG 3350, 0.1 M BisTris pH 5.5, 0.2 M ammonium acetate.

To avaluate if the CysA2 was stable or partially folded, the thermal stability was monitored by the CD signal at 222 nm during the increasing of the temperature from 20 °C to 100 °C (Fig. 3c). The calculated temperature of melting (T*m*) was equivalent to 58.3 °C indicating the protein was stable. The presence of thiosulfate or cyanide did not affect this result and the protein exhibited irreversible thermal unfolding. The behavior of CysA2 in absence of co-substrates, presence of cyanide, thiosulfate and cyanide +thiosulfate also was avaluated by dynamic light scattering (Fig. 3d). In this essay, the protein in absence of co-substrates showed a hydrodynamic radius (*rh*) of 3.8 nm and a calculated molecular mass of 72 kDa, which was equivalent to a dimer in solution (Fig. 3d, 10 min). With addition of the substrates CysA2 showed a time-dependent *rh* reduction that reached a minimum of 3 nm and a molecular mass of 44 kDa, which was followed by reestablishment of the original parameters (Fig. 3d). Since CD spectra under the same conditions revealed only subtle differences with respect to untreated CysA2 with its co-substrates (Fig. 3a), it might be suggested that the catalytic mechanism of the enzyme involves dissociation and reassociation of its monomers.

### Enzymatic activity of CysA2 shows it is capable to use both thiosulfate and 3-mercaptopyruvate as substrate

**Figure 4:**
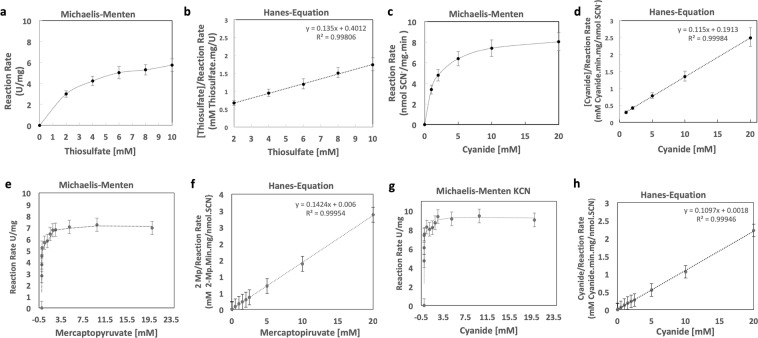
Kinetic analysis of CysA2 using thiosulfate, 3-mercaptopyruvate and cyanide as co-substrates. The thiosulfate:cyanide sulfurtanserase activity or mercaptopyruvate:cyanide sulfurtanserase activity of the enzyme CysA2 was evaluated via a modification of the Sorbo method^88^. The reaction mixture consisted of 100 mM Tris-HCl, pH 8.0, 50 mM thiosulfate or 50 mM Mercaptopyravate, in a total volume of 600 μL. The mixture was preincubated at 37 °C for 10 minutes, and the reaction was started by the addition of 50 mM KCN the absorbance of the supernatant was measured at 460 nm. One unit of sulfurtransferase activity was defined as 1 nmol of SCN- formed every minute. (**a**) Enzimatic cinetic to thiosulfate. Km of 2.97 mM +/− 0.05 (SEM) and Vmax of 7.40 U/mg +/− 0.72 U/mg (SEM). Error Bar = Standard deviation of the mean (SEM). (**b**) Linearization by Hanes-Equation to enzymatic assay to thiosulfate substrate. (**c**) Enzymic kinetics to KCN, Km of 1.66 mM +/− 0.05 (SEM) for reaction thiosulphate. Error Bar = Standard deviation of the mean (SEM). (**d**) Linearization by Hanes-Equation to enzymatic assay to cyanide substrate. (**e**) Enzimatic cinetic to Mercaptopyruvate. Km of 7.02 mM +/− 0.05 (SEM) and Vmax of 0.0461 U/mg +/− 0.0012 U/mg (SEM). Error Bar = Standard deviation of the mean (SEM). (**f**) Linearization by Hanes-Equation to enzymatic assay to mercaptopyruvate substrate. (**g**) Enzymic kinetics to KCN. 9.11 mM +/− 0.05 (SEM) for reaction 3-mercaptopyruvate. Error Bar = Standard deviation of the mean (SEM). (**h**) Linearization by Hanes-Equation to enzymatic assay to cyanide substrate.

Enzymatic analysis of CysA2 showed that the enzyme was able to use both thiosulfate and 3-mercaptopyruvate as substrates (Fig. 4a–h, respectively). Assays defined the enzymatic activity of CysA2 through the kinetic parameters observed in the Michaelis-Menten constant (K_*m*_) and the maximum velocity of enzymatic catalysis (V_*max*_) determined using the Hanes equation. The K_*m*_ for thiosulfate was 2.97 mM +/− 0.05, while the K_*m*_ for cyanide was 1.66 mM +/− 0.03. V_*max*_ for thiosulfate was 7.40 mmol SCN^−^/mg.min +/− 0.72 mmol SCN^−^/mg.min, while V_*max*_ for cyanide was 8.69 mmol SCN^−^/mg.min +/− 0.93 mmol SCN^−^/mg.min (Fig. 4a–d). An unpaired T test was used to compare the two maximum velocities obtained and no significant statistical difference was found. Enzymatic kinetics experiments using 3-mercaptopyruvate showed an additional ability of the enzyme to also utilize this substrate, differently from what is expected for rhodaneses. The obtained K_*m*_ and V_*max*_ for 3-mercaptopyruvate were respectively calculated in 7.02 mM +/− 0.05 mmol SCN^−^/mg.min and 46.1 +/− 0.0012 mmol SCN^−^/mg.min. For cyanide, the results were K_*m*_ of 9.11 +/− 0.05 mM and V_*max*_ of 0.0164 +/− 0.009 mmol SCN^−^/mg.min (Fig. 4e–h).

The efficiency of the catalysis of cyanide, thiosulfate and 3-mercaptopyruvate by CysA2, was evaluated in comparison with kinetic parameters obtained for other sulfurtransferases (Table 1). The results indicated that low concentrations of the substrate are not able to fully occupy the active site of the enzyme CysA2, as showed by the relatively low K_*m*_, especially when considering the same parameters obtained for Rhobov protein, a model of rhomboid structure and function. Once the rate of reaction is directly related to site occupancy, we have a V_*max*_ with a value in the order of nmolar while other enzymes show units in the μmolar range. In fact, it is very unlikely that the natural substrates (*in vivo*) of these enzymes be cyanide and thiosulfate due to the high toxicity of these molecules to the cell. This feature might explain the low capacity of the conversion of the substrates to the expected product^15,32^. Although CysA2 conserves the functional motifs of thiosulfate sufurtransferases, its kinetic activity against thiosulfate was very low, suggesting that *in vivo*, different substrates might be used. The ability of CysA2 to use 3-mercaptopyruvate as a thiol group donor was also analysed. Protein activity was monitored in a constant concentration of cyanide potassium (5 mM) revealing K_*m*_ and V_*max*_ values for mercaptopyruvate of 7.02 mM and 0.461 μmol/min.mg, respectively.

**Table 1:** Kinetic parameters obtained for *M. tuberculosis* CysA2 in comparison with TSTs and MSTs previously characterized in the literature.

Enzymes TST	Enzyme activity				Reference
	K_m_ cyanide (mM)	K_m_ thiosulfate (mM)	K_m_ 3-mp (mM)	Vmax (µmol.min^−1^.mg^−1^)	
*Mycobacterium tuberculosis* CysA2 (Mtu0100)	1.66	2.97	—	0,008 × 10^−3^	This work
*Arabdopsis thaliana* (ATIG16460)	—	7.2	5.4	—	^58^
*Rattus norvergicus* (Rno25274)	—	4.4	2.6	1.1 × 10^4^	^59^
*Bos taurus* Rhobov (Bta280946)	0.063	7.0	—	600	^12^
*Azotobacter vinelandii* RhdA (AvCA607450)	8.7	1.0	—	1250	^30^
*Pseudomonas aeruginosa* RhdA (PaePA4956)	16	7.4	—	815	^16^
*Fusarium solani* (Necha2_57025)	2	—	—	0.083	^17^
*Trichoderma harzianum* (COG2897)	7	—	—	0.069	^17^
*Mycobacterium tuberculosis* CysA2 (Mtu01000)	9.11	—	7.02	0,0461 × 10^−3^	This work
*Escherichia coli* SseA (Ecob2521)	1.25	—	8.34	—	^60^
*Homo sapiens* (Hsa4357)	130	—	350	417	^37^
*Arabidopis thaliana* (ATIG79230)	—	—	11	2.4 × 10^3^	^58^
*Leishmania major* (LMJF050970)	—	10.9	345	0.2	^32^
*Leishmania mexicana* (LMXM050970)	—	20.8	345	—	^32^
*Rattus norvegicus* (Rno192172)	—	6.2 × 10	1.2	—	^58^

MST1_*Arabidopsis thaliana* (AT1G79230); MST2_*Arabidopsis thaliana* (AT1G16460); TST_*Rattus norvegicus* Rno25274); MST *Rattus norvegicus* (Rno192172); Rhobov_*Bos Taurus* (Bta280946); RhdA_A*zotobacter vinelandii* (AvCA607450); RhdA_*Pseudomonas aeruginosa* RhdA (PaePA4956); MST_*Leishmania major* (LMJF050970); MST_*Leishmania mexicana* (LMXM050970); TST_*Trichoderma harzianum* (COG2897)*;* TST_*Fusarium solani* (Necha2_57025). (—) not identified.

**Figure 5:**
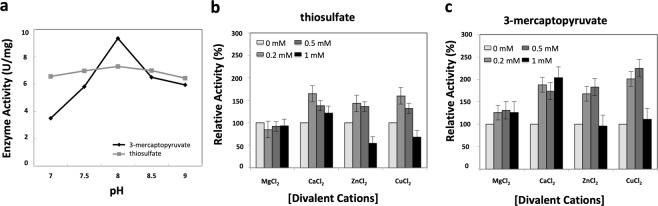
Influence of divalent cations and pH in the activity of *M. tuberculosis* CysA2. (**a**) Effect of CaCl_2_, CuCl_2_, MgCl_2_ and ZnCl_2_ in the CysA2 activity using thiosulfate (gray bars) and 3-mercaptopyruvate (black bars) as substrates. The activity of the enzyme in the absence of metals was taken as the reference unit. Error Bar = 95% confidence interval. One-Way ANOVA p-value < 0.001. (**b**) The effect of pH also was evaluated using 1 mg/mL of purified CysA2 in 100 mM Tris-HCl buffer (pH 7.0, 7.5, 8.0, 8.5 and 9.0), 30 mM KCN and 30 mM of thiosulfate or 3-mercaptopyruvate. The results represent the mean of three independent experiments for each substrate. Error bars confidence interval 95%. One-way ANOVA p-value < 0.001.

Once the results showed low values for activity both as TST and MST, we also evaluated the pH and cations influence on CysA2 activity (Fig. 5). As for the effect of pH on CysA2 activity, it is observed that the enzyme is capable of operating efficiently over a wide pH range (7.0 to 9.0) (Fig. 5a). Rhodaneses of other species also have an optimal pH range around 8 and even more basic pH^31,33^.

The effect of cations on CysA2 activity was evaluated since classical studies with the bovine rhodanese showed a divalent cation-binding region in its structure^34^. The CysA2 analyses were performed in increased concentrations of Cu^2+^, Zn^2+^, Mg^2+^ and Ca^2+^. The results showed that concentrations of 0.2 to 0.5 mM of cations have positive effect on the CysA2 enzymatic activity but not 1 mM (Fig. 5a,b). Exception was evidenced for CysA2 in presence of 3-mercaptopyruvate and 1 mM CaCl_2_ that showed a significant increasing in the activity. In general, the effect of cations on CysA2 activity was more evident with 3-mercaptopyruvate than thiosulfate.

### CysA2 can bind to pulmonary mouse cells and has the ability to activate dendritic cells and macrophages

**Figure 6:**
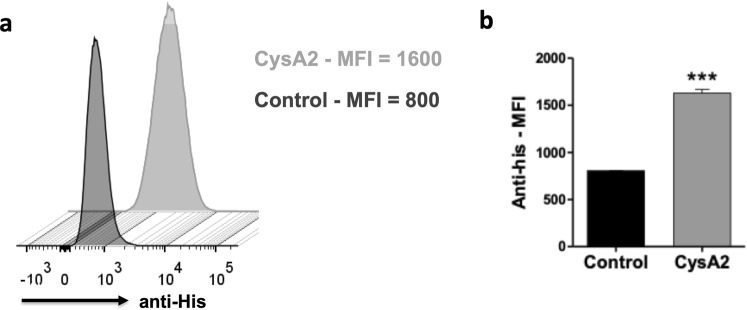
The CysA2 protein can bind to pulmonary mouse cells. The protein-TC-1 cells binding assays were performed with purified CysA2 protein. After blocking Fcγ receptors, cells were treated with CysA2 and further stained with purified anti-His-tag IgG monoclonal antibody. The IgG molecule was detected by the anti-IgG-PE. (**a**) Flow cytometry data analysis by histograms, which measure and compare only a single parameter on the X-axis, being the amount of anti-his antibody fluorescence from the control group (only TC-1 cells) versus the CysA2 group (TC-1 cells treated with CysA2). (**b**) Representative graph of the median of fluorescence intensity comparing the control group versus the CysA2 group. The analysis was performed in LSR Fortessa flow cytometers (BD Bioscience), and data were analyzed by FlowJo software (version 10.0.7 - Tree Star). Experiments were performed twice, in triplicate. MFI - median of fluorescence intensity; ***p < 0.001.

**Figure 7:**
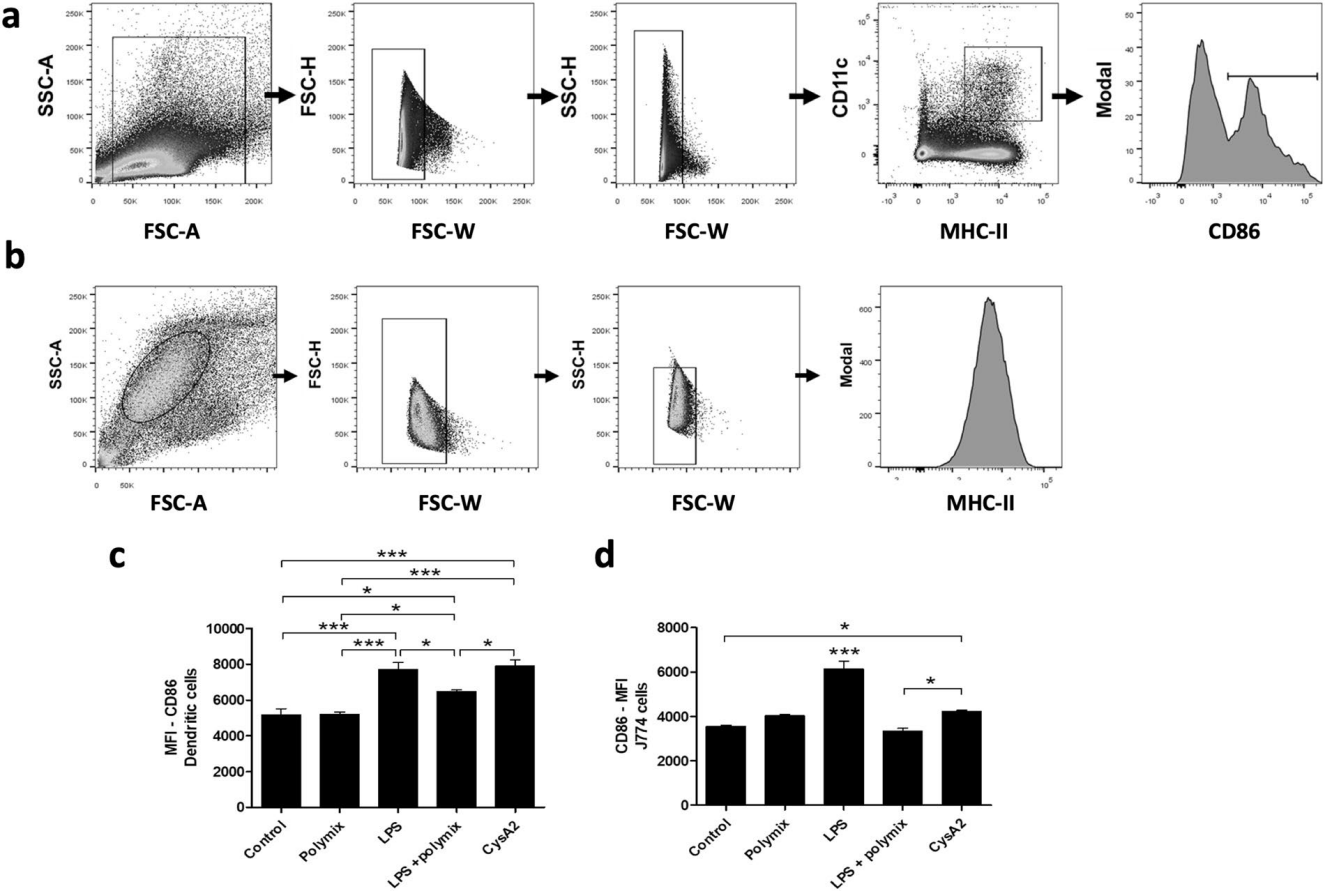
CysA2 is an immunogenic protein capable of activate dendritic cells and macrophages. Murine dendritic cells and macrophages were incubated with CysA2 for further CD86 expression analysis. Polymyxin was used to neutralize any LPS contamination on CysA2 protein. LPS was used as a positive control of immune cells activation. (**a**) The gate strategy for the analysis of dendritic cells activation. Initially, side scatter area versus forward scatter area density plots (SSC-A vs FSC-A) was used to identify the cell population of interest (inside the gate) and exclude the cellular debris. Subsequently, cellular doublets were excluded from analysis by forward scatter height (FSC-H) versus forward scatter width (FSC-W), followed by the side scatter height (FSC-H) versus side scatter width (FSC-W) parameters. The cells of interest were always inside the gate. Dendritic cells were separated from splenic single cells after cells gated according to cell type specific markers, being the CD11c^+^ and MCH-II^+^ molecules expression. Dendritic cells activation was evaluated by the expression the CD86^+^ co-stimulatory molecule. (**b**) The gate strategy of J774 cells. Cellular debris and cellular doublets were excluded as describe above. Murine J774 cells activation was evaluated after single cells gated by the expression of CD86^+^. CysA2 was able to induce CD89 upregulation on (**c**) dendritic cells and (**d**) J774 macrophages. (**e**) By SDS-PAGE followed by western-blotting analysis, we observed that CysA2 is probably binding to U937 human macrophages, as well as could also be phagocytized by them. (A-D) The analysis was performed in LSR Fortessa flow cytometers (BD Bioscience), and data were analyzed by FlowJo software (version 10.0.7 - Tree Star). All experiments were performed twice, in triplicate. MFI - median of fluorescence intensity; Mɸ - macrophages. *p < 0.05; ***p < 0.001.

The identification and presentation of antigen by target cells, mostly antigen presenting cells (APCs), involves the expression of specific molecules. Exogenous pathogens proteins are usually processed and presented via MHC class-II molecules by APCs, which in turn became activate and upregulate the expression of co-stimulatory molecules, as CD86^35,36^. Additionally, pathogens can be recognized by specific receptors from epithelial cells. Therefore, to understand some mechanisms involved in the *M. tuberculosis* recognition by target cells, we evaluate the immunogenic properties of CysA2 and explored its interaction with epithelial and immune cells. Since the target infection site of the *M. tuberculosis* is the lung, we first evaluated if CysA2 was able to bind to the TC-1 cells (Fig. 6), a transformed pulmonary cell from C57BL-6 mouse. Binding of CysA2 to TC-1 cells was assess by its labeling with an anti-histidine monoclonal antibody followed by a secondary labeling with an anti-IgG (Fig. 6a). Interestingly, in our experimental settings, CysA2 showed a great ability to bind to TC-1 cells (Fig. 6b), highlighting the possibility of its role in bacterial adhesion and, consequently, its importance in the *M. tuberculosis* recognition by epithelial cells. Furthermore, using the purified protein, we also evaluated the ability of CysA2 in activating two important antigens presenting cells (APCs), dendritic cells and macrophages (Fig. 7). The *ex-vivo* dendritic cells belonging to total the heterogeneous population of splenic cells from C57BL/6 mice were evaluated by multiple parameters after immunophenotyping (Fig. 7a). The murine macrophage cell line J774 activation was evaluated after gated as single cells for further analysis of the expression of the co-stimulatory molecule CD86 (Fig. 7b). Fortunately, CysA2 showed to be immunogenic, once it was able to activate the expression of CD86 in both murine dendritic cells (Fig. 7c) and murine J774 macrophages (Fig. 7d). Interestingly, the potential of CysA2 to activate immune cells was more pronounced in dendritic cells than J774 macrophages, since CD86 expression in this experimental group was similar to the positive control group (LPS). The co-stimulatory molecules CD40 and CD80 were not upregulated upon CysA2 treatment. These results clearly state that CysA2 stimulates the cellular immune response, responsible for antigen presentation and/or microbial degradation.

## Discussion

The few literature data that may be associated with *M. tuberculosis* CysA2 have shown that this enzyme plays an important role in the physiology of bacillus. Its three-dimensional structure reveals the classical sulfurtransferase/rhodanese domains, characteristics of enzymes importantly active in the metabolism of different organisms, as well as a shape with pockets and cavities, which make it an excellent target for the development of inhibitors using, for example, the methodology of fragment based drug discovery. In addition, its potential as an immunological agent draws attention to the use of this protein as a vaccine epitope or even for diagnosis, once the current diagnostic methods exhibit low sensitivity, require a long time to perform the microbiological cultures and low specificity for *M. tuberculosis*. In this work, we described the functional features that set it as sulfurtransferase and performed biophysical characterization to define the kinetic activity. Moreover, we explored its potential as immunogenic target.

CysA2 shares high sequence similarity with TSTs and its capability to use 3-mercaptopyruvate as substrate was shown, although the enzymatic parameters were significatively better in the presence of thiosulfate as substrate. On the other hand, the K_*m*_ values in the order of millimolar show that both thiosulfate and 3-mercaptopyruvate should not be the physiological substrates. Moreover, *M. tuberculosis* CysA2 presented low activity when compared to other TSTs and also MSTs. Considering these results and comparing to similar studies with proteins from sulfurtransferase family, it was found that while the enzymes of the sulfurtransferase group are specific for the type of donor in the thiol group, other enzymes of this class seem to show some promiscuity for the donor type^30,37,38^. In MST1 and MST2 enzymes of *A. thaliana* that are able to catalyze both thiosulfate (TST) and 3-mercaptopyruvate (MST), the cysteine in the “CR(Y/R)” motif is essential for the enzyme’s activity in the presence of any substrate. Different results were found for sulfurtransferases from men and cattle, the latter considered as a model of TST^37^. In these organisms the enzymes showed specificity for each of the substrates, being well defined as MST or TST. Similar to the enzyme identified in rat that showed ability to transit between the two substrates, *M. tuberculosis* CysA2 also performed as MST and TST, with better activity with thiosulfate as substrate. In the case of protozoa, *Leishmania major*, *Leishmania mexicana* and *Trichoderma harzianum*, also eukaryotes, the behavior of the enzymes is also dubious, with preference for substrates defined on characteristics signatures^17^.

The sequence of the catalytic site of all the MSTs analyzed by the kinetic activity in the literature presents high conservation of the amino acids and we can not identify with this analysis the permissive point for the use of one or the other substrate. In sulfurtransferases, alpha-helix macropoles surrounding the catalytic cysteine are important to create a redox environment that will support hydrogen bonds between sulfur atom of a persulfide and surrounding amino acids stabilizing catalytic intermediates. As for the effect of pH on CysA2 activity, it was observed that the enzyme is able to operate efficiently over a wide pH range with optimum activity at pH 8.0, which is in accordance with rhodaneses from other species^33^.

Regarding the *M. tuberculosis* pathogenesis, *M. tuberculosis* can infect epithelial cells both *in vitro* and *in vivo*^39–42^. It is believed that the bacillus interactions during infection may play a relevant role during the onset of the infectious process and that they may influence the immune response of the host^43,44^. Tuberculosis is basically a lung disease, the lung being the entry point of the bacillus and the main site of infection manifestation. Interestingly, although CysA2 belongs to a cytoplasmic pathway, it has been identified by mass spectrometry in other cell regions such as cell wall, membrane and *M. tuberculosis* culture supernatants^10,45–47^. Concerning this last feature, it is likely that this protein can be recognized by the immune system. During bacterium pathogenesis, *M. tuberculosis* is phagocytosed by alveolar macrophages and dendritic cells that migrate through the lymphatic system and simultaneously can penetrate the lung parenchyma, attracting other macrophages and initiating the inflammatory focus^48^. Unraveling other properties of CysA2, this is an immunogenic protein, capable of being recognized and phagocytosed by dendritic cells and macrophages, leading to the upregulation of co-stimulatory molecules. Since CysA2 was able to bind to a pulmonary cell line, we hypothesized that this protein may be important to bacterial adhesion and/or recognition by host cells, suggesting a role during bacillus infection. Furthermore, the binding of CysA2 to TC-1 cells corroborates with previous studies that suggested its relevance as a serological marker for detection of tuberculosis in patients^8^.

Given that CysA2 is secreted only under special culture conditions, that it has high levels of expression in infected patients^44^ and the redundancy in the genome of *M. tuberculosis*^4^, CysA2 might be involved in the formation of such lipid rafts and could be the candidate that would play a relevant role in the process of infection of lung epithelial cells. Therefore, the CysA2 protein may behave as a good candidate for tuberculosis diagnosis, since it was recognized in serum from patients infected with *M. tuberculosis*^8^. Faced with these different properties of CysA2, this molecule might be a moonlighting protein, performing more than one function^49^. Many of moonlighting proteins have as their main characteristic, the activity in enzymatic catalysis and aggregation, and secondary, role in signal transduction, transcriptional regulation and apoptosis^50^. Furthermore, the presence of a gene duplication (*cysA3*) highlights the relevance of the respective proteins to the cell.

In conclusion, the dataset presented in this work consist of the first characterization of *M. tuberculosis* CysA2 evidencing relevant immunological features for its exploration in assays to reach a significant pool of proteins for diagnosis at the various stages of TB infection and manifestation. Moreover, due the importance of the protein for the bacillus, its kinetic activity and three-dimensional structure, which is highly drugable, CysA2 is an interesting target for the development of inhibitors.

## Methods

### Bioinformatics analyses

CysA2 amino acid sequence was obtained in the TubercuList database (http://tuberculist.epfl.ch). Searching for orthologues in different species and related proteins with solved three-dimensional structures was performed using the BlastP tool (http://blast.ncbi.nlm.nih.gov) against the non-redundant protein sequence database (nr) and PDB, respectively. Amino acid sequences alignments were performed using ClustalW server (http://www.genome.jp/tools-bin/clustalw). Superimposition of the three-dimensional structures from CysA2 and orthologues was performed using in COOT^51^ and analyzed in Pymol^52^.

### Gene cloning, protein expression and purification

*cysA2* gene was amplified by PCR using the *M. tuberculosis* genomic DNA as template and the oligonucleotides 5′-GAATTCATGGCACGCTGCGATGTCCTG-3′ (FcysA2) and 5-CTCGAGTCA GCTTCCCAACTCGATCGG-3′(RcysA2). Restriction sites for enzymes *Eco*RI and *Xho*I (underlined) were introduced in the forward and reverse oligonucleotides, respectively. The digested PCR product was cloned into pET-28a (Novagen, LA, CA, EUA), dowstream of the encoding His_6_tag sequence and checked by DNA sequencing. The plasmid was introduced in *E. coli* BL21(DE3) by chemical transformation. Protein expression was obtained after growth of the cells up to mid-log phase (OD_600_ ~ 0.6) at 37 °C in LB medium with kanamycin (30 μg/ml) and induction by 4 hours after addition 0.5 mM isopropyl thio-β-D-galactopyranoside (IPTG). Soluble extracts were obtained after sonication of the cells (8 cycles x 15 *s* in a Brandson Sonifier 450) in lysis buffer (25 mM Tris-HCl pH 8.0, 500 mM NaCl, 100 μM PMSF) and centrifugation at 14.000 *g* for 40 minutes. The supernatant was immediately submitted for immobilized metal affinity chromatography (IMAC) using a column HiTrap IMAC HP (Qiagen, Boston, MA, EUA) and eluted with an imidazole gradient (0–500 mM). Eluted fractions were analyzed by 15% (w/v) SDS-PAGE after Coomassie blue staining. Pure fractions of CysA2 were concentrated and submitted for size-exclusion chromatography (SEC) using a Superdex 200 16/60 gel filtration column (GE Healthcare, Chicago, Illinois, EUA) calibrated with a HMW Gel Filtration Calibration Kit (Pharmacia Biotechnology Inc., Piscataway, NJ) at a flow rate of 1 ml/min with the equilibration buffer (25 mM Tris-HCl pH 8.0, 150 mM NaCl). CysA2 samples were pooled and extensively dialyzed against 20 mM Tris-HCl pH 8.0 at 4 °C and the final protein concentration was estimated using the extinction coefficient ε_280nm_ = 60.515.

### Dynamic light scattering analysis

Experiments were performed using a DynaPro 810 system (Wyatt Technology Corporation, Santa Barbara, CA, EUA) and DYNAMICS v.6.3.40 software. DLS experiments were carried out in a wide range of protein concentration (1–10 mg/mL) to analyze aggregation and to estimate CysA2 hydrodynamic radius. DLS measurements (100 measurements at 5 *s* intervals) were conducted at 291 K and the hydrodynamic radius (Rh) was extrapolated from the translational diffusion coefficient (Dt) using the Stokes–Einstein equation^53^.

### Circular dichroism analysis

Far-UV circular dichroism (CD) spectra of the enzyme in presence and absence of substrates were recorded on a Jasco J-810 spectropolarimeter (Jasco International Co., Tokyo, Japan). CD measurements were carried out in a 1 mm quartz cuvette using the wavelength range of 200–260 nm. The protein concentration was set to 5 μM in Tris-HCl 20 mM pH 8.0 with 5 mM of the substrats thiosulfate, 3-mercaptopyruvate and KCN. The data collection parameters were set to scan rate of 50 nm/min, response time of 4 *s*, sensitivity of 100 mdeg, scan step of 0.5 nm and 20 accumulation rounds. To study the effect of temperature on CysA2 structure, the samples were heated from 10 °C to 103 °C. The data collection parameters were exactly as described before, including the heating rate of 1 °C/min and delay time for spectrum collection of 60 *s*. The reversibility of the temperature effect was monitored from 103 °C to 20 °C using the same parameters described above. Baseline subtraction, smoothing and data normalization were carried out using the graphical software ORIGIN (http://www.originlab.com/). The CD data are shown as mean residue ellipticity units (deg.cm^2^.dmol^−1^) and evaluated by deconvolution of the CD spectrum using the DichroWeb server^54^.

### Kinetic assays

The CysA2 sulfurtransferase activity was measured following the SCN- formation colorimetrically determined according to Sorbo’s method^55^ using thiosulfate or 3-mercaptopyruvate as sulfur donors and cyanide as sulfur acceptor. The kinetic parameters of CysA2 were obtained with 4 μg of protein in Tris-HCl 0.1 M pH 8.0 buffer with 50 mM KCN and 40 mM thiosulfate (STS assay) or 30 mM KCN and 30 mM 3-mercaptopyruvate (MTS assay). The reaction mixtures were incubated at 37 °C for 30 minutes and stopped with addition of 0.5 ml formaldehyde (15%), followed the addition of 2.5 ml of ferric nitrate (1%). The proportion of ferric ion complex and thiocyanate was measured spectrophotometrically at 460 nm using a spectrophotometer Epoch2 (Biotek Instruments, Vermont, USA). The steady-state kinetics was evaluated by Michaelis-Menten equation^56^. The influence of the pH in the enzymatic activity was checked in 100 mM Tris-HCl pH 7.0 and 9.0. The behavior of the enzyme was also evaluated in absence and presence of 0.2 mM, 0.5 mM and 1 mM of the different ions: CaCl_2_, CuCl_2_, MgCl_2_, or ZnCl_2_ and 30 mM of substrate.

### Pulmonary TC-1 mouse cells binding assay

The binding assays were performed as previously described^35^. Briefly, TC-1 cells (4 × 10^5^/well)^57^, generously provided by Dr. T. C. Wu from Johns Hopkins University, Baltimore, USA, in 2002, were incubated with CD16/CD32 antibodies (FcBlock, BD Biosciences) for 15 min on ice. Then, 5 µg/mL of CysA2 were added to cells, followed by an additional incubation of 40 min on ice. After washing cells with MACS buffer [phosphate-buffered saline (PBS), pH 7.2, 0.5% bovine serum albumin (BSA), and 2 mM EDTA], cells were incubated with anti-His-tag IgG monoclonal antibody (Thermo Fischer) for 40 min on ice. Then, after washing cells twice, TC-1 cells were stained with anti-IgG-PE antibody (Jackson ImmunoResearch) for 30 min on ice. Finally, cells were washed twice and resuspended in MACS buffer for further analysis in LSR Fortessa flow cytometers (BD Bioscience). Data were analyzed by FlowJo software (version 10.0.7 - Tree Star). Experiments were done twice, in triplicate.

### Murine dendritic cells and macrophages cell line activation assay

Wild type (WT) C57BL/6 mice were purchased from the Faculty of Veterinary Medicine of the University of São Paulo. Spleen were removed and macerated with the aid of a syringe plunger, suspended in RPMI 1640 medium supplemented with 10% FBS and 50 U/mL penicillin/streptomycin (R10), filtered in a 70 μm cell strainer (Easy strainer Greiner Bio One) and treated with ACK lysis buffer for the removal of red blood cells. Then, total splenic cells were washed and resuspended in MACS buffer for further staining. J774 murine macrophages cell lines were cultured in R10 medium at 37 °C and 5% CO_2_. The J774 cell line (J774A.1) is a murine (BALB/cN strain) cell derived from mouse ascites with macrophage properties from an already-existing cell collection from the Vaccine Development Laboratory, University of São Paulo, São Paulo, SP, Brazil. For immune cells (total splenic cells and J774 cells) activation, 3 × 10^6^ cells/mL were seeded into a 15 mL conic tube. CysA2 protein was previously treated with polymyxin to inactivate LPS contamination^36^. Pre-treated CysA2 protein was added to immune cells at a final concentration of 5 µg/mL. Cells were incubated for 48 hours in R10 medium at 37 °C and 5% CO_2_. Then, cells were washed twice with MACS buffer and placed into a 96-well U-bottom plates for further staining. The following mAbs were used for the immunophenotyping of murine dendritic cells: anti-CD11c-PE (#553802, BD Pharmingen), anti-MHC-II-FITC (#553605, BD Pharmingen). For the analysis of immune cells activation, we used the anti-CD86-APC (#558695, BD Pharmingen). Cells were stained as previously described^32^. Cells were acquired by LSR Fortessa (BD Biosciences) flow cytometer and data were analyzed using the FlowJo software. All the animal experiments were performing according to the procedures for manipulation and euthanasia of mice and they were approved by the Ethics Committee for Animal Experimentation (CEUA), Institute of Biomedical Sciences, University of São Paulo (protocol number 050/2014), following rules from the National Council for Control of Animal Experimentation (CONCEA, Ministry of Science and Technology, Brazil).

### Interaction of CysA2 to human macrophagic cells

The human U937 lineage monocytes (ATCC CRL-1593.2^TM^) belonging to the cell collection from the Vaccine Development Laboratory, University of São Paulo, São Paulo, SP, Brazil, were differentiated into macrophages with PMA and treated with 0.1 mg/ml recombinant CysA2 diluted in R10 medium for 5 hours. After 6 washes with PBS, the cells were lysed, fractionated by SDS-PAGE. 50 μg of total proteins from each group condition were analyzed by Western blot using anti-6xHis as a probe.

### Ethics statement

The procedures for manipulation and euthanasia of mice were carried out in accordance with relevant guidelines and regulations and were approved by the Ethics Committee for Animal Experimentation (CEUA) (protocol number 050/2014), Institute of Biomedical Science, University of São Paulo, following rules from the National Council for Control of Animal Experimentation (CONCEA, Ministry of Science and Technology, Brazil).

## Supplementary information

Supplementary information

## Data Availability

The authors have declared that all data are fully available without restriction.
