# Supplementary material for: Mycobacterium tuberculosis CysA2 is a dual sulfurtransferase with activity against thiosulfate and 3-mercaptopyruvate and interacts with mammalian cells
Source: Sci Rep. 2019 Nov 14;9:16791. doi: 10.1038/s41598-019-53069-6 (PMC6856128; doi:10.1038/s41598-019-53069-6)
Supplement: Supplementary file 1 — Supplementary information [file 41598_2019_53069_MOESM1_ESM.docx]

**Supplementary Information**

***Mycobacterium tuberculosis* CysA2 is a dual sulfurtransferase with activity against thiosulfate and 3-mercaptopyruvate and interacts with mammalian cells**

**Meza AN, Cambui CCN, Moreno ACR, Fessel MR, Balan A**


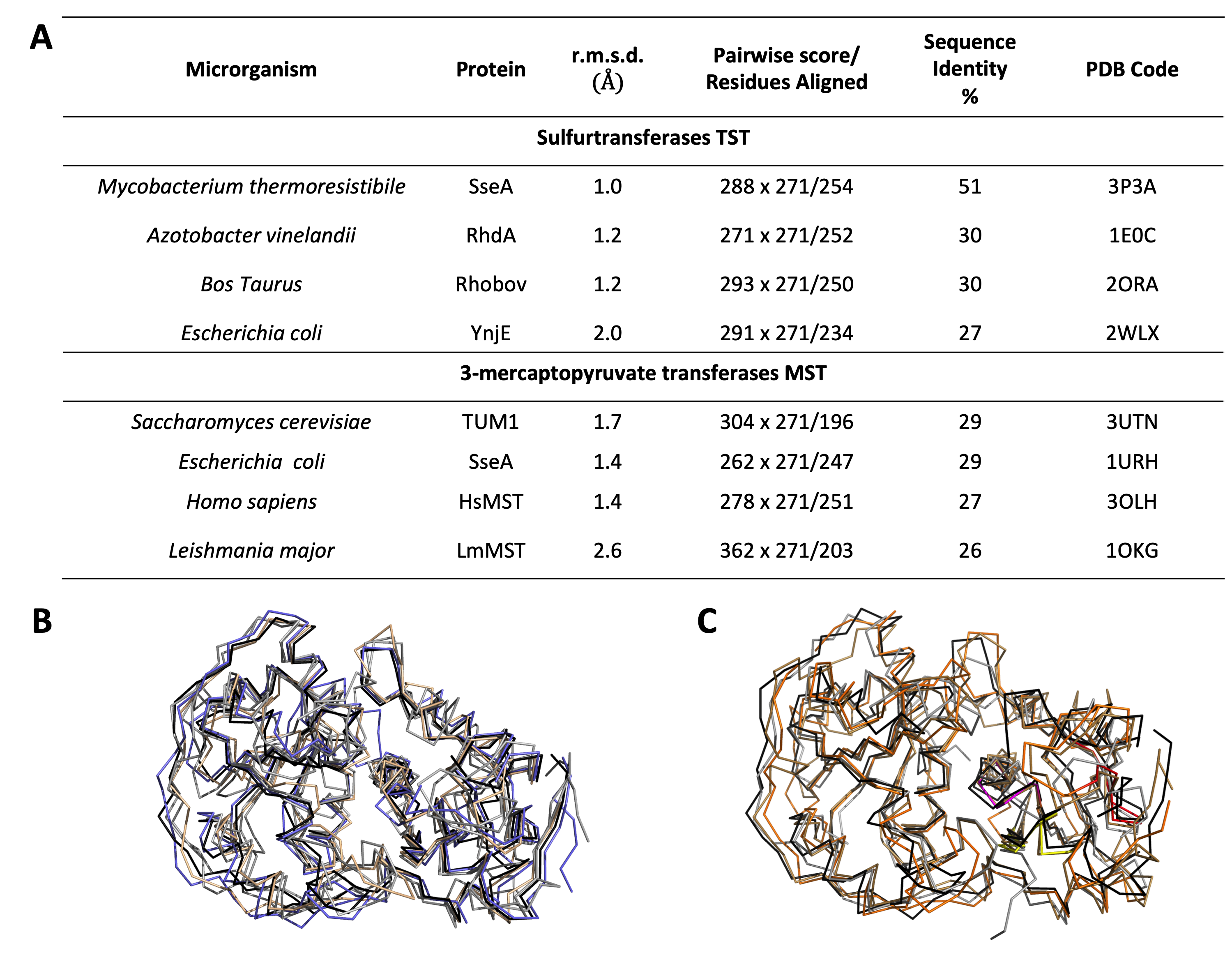


**Figure S1.** Comparison of *M. tuberculosis* CysA2 with the proteins identified after the Blastp X PDB (Protein Data Bank). (**a**) List of characteristics of the thiosulfate sulfurtransferases (TST) and 3-mercaptopyruvate transferases (MST) identified in comparison with CysA2. (**b**) Structural alignment of *M. tuberculosis* CysA2 structure with the TSTs and (**c**) with the MSTs. The structural alignment was performed using the secondary structure matching in COOT. CysA2 structure is represented in black ribbon.


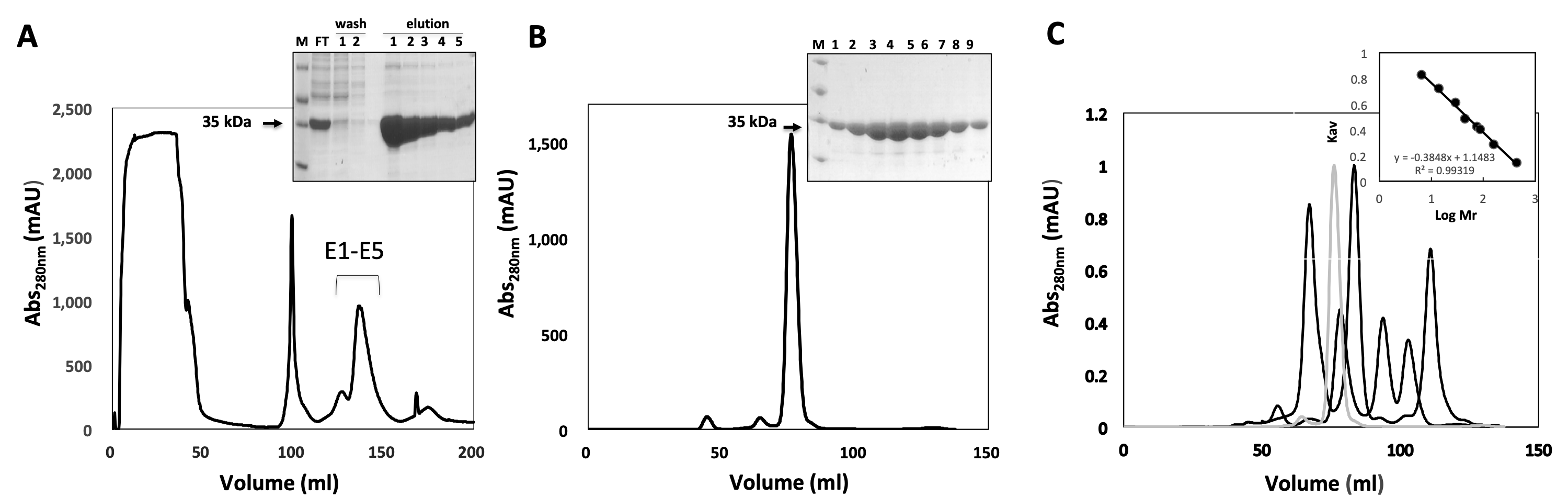


**Figure S2.** Overexpression and purification steps of *M. tuberculosis* CysA2. (**a**) SDS-PAGE 12% stained with Coomassie brilliant blue. M = molecular mass markers, FT = Flow through, E1-E5 = eluted fractions with 140 mM Imidazole (1 to 5). (**b**) CysA2 size exclusion chromatogram. The inset shows the SDS-PAGE 12% stained with Coomassie brilliant blue. 1-9, eluted fractions corresponding to the high peak. (**c**) Analytical size-exclusion chromatography performed on a HiLoad 16/60 Superdex 200 pg column. The elution pattern of CysA2 is shown in gray and the correspondent to the standard proteins in black. Ferritin (440 kDa), aldolase C, conalbumin (77 kDa), ovalbumin (45 kDa), carbonic anhydrose (29 kDa), ribonucleaseA (13.7 kDa) and aprotinina (6.5 kDa). The molecular mass estimated for CysA2 based on the calibration curve (inset) was 76.1 kDa, which is compatible with the theoretical mass of a dimer (37 kDa). Kav, partition coefficient; Mr, relative molecular mass.
